# Smart Ship Draft Reading by Dual-Flow Deep Learning Architecture and Multispectral Information

**DOI:** 10.3390/s24175580

**Published:** 2024-08-28

**Authors:** Bo Zhang, Jiangyun Li, Haicheng Tang, Xi Liu

**Affiliations:** 1 China Coal Research Institute Corporation, Beijing 100013, China; zb_ccric@163.com; 2 School of Automation and Electrical Engineering, University of Science and Technology Beijing, Beijing 100083, China; thc576495032@163.com (H.T.); m202210556@xs.ustb.edu.cn (X.L.); 3 Key Laboratory of Knowledge Automation for Industrial Processes, Ministry of Education, Beijing 100083, China

**Keywords:** ship draft reading, dual-flow architecture, multispectral image, computer vision

## Abstract

In maritime transportation, a ship’s draft survey serves as a primary method for weighing bulk cargo. The accuracy of the ship’s draft reading determines the fairness of bulk cargo transactions. Human visual-based draft reading methods face issues such as safety concerns, high labor costs, and subjective interpretation. Therefore, some image processing methods are utilized to achieve automatic draft reading. However, due to the limitations in the spectral characteristics of RGB images, existing image processing methods are susceptible to water surface environmental interference, such as reflections. To solve this issue, we obtained and annotated 524 multispectral images of a ship’s draft as the research dataset, marking the first application of integrating NIR information and RGB images for automatic draft reading tasks. Additionally, a dual-branch backbone named BIF is proposed to extract and combine spectral information from RGB and NIR images. The backbone network can be combined with the existing segmentation head and detection head to perform waterline segmentation and draft detection. By replacing the original ResNet-50 backbone of YOLOv8, we reached a *mAP* of 99.2% in the draft detection task. Similarly, combining UPerNet with our dual-branch backbone, the *mIoU* of the waterline segmentation task was improved from 98.9% to 99.3%. The inaccuracy of the draft reading is less than ±0.01 m, confirming the efficacy of our method for automatic draft reading tasks.

## 1. Introduction

In the era of globalization, international trade is on the rise, with maritime transportation emerging as a primary means for the import and export of goods. For instance, the global trade volume of dry bulk shipping reached 5.508 billion metric tons in 2023. To weigh low-value or difficult-to-weigh solid bulk cargo, a ship’s draft reading is a convenient and popular method that is based on Archimedes’ principle. It generally requires precise reading due to its direct impact on economic benefits. As an example, a 1 cm error by manually observing the draft line of a 50,000-ton bulk carrier can result in an 80-ton error, valued at USD 40,000 at USD 500/ton. Thus, improving the accuracy of ship draft reading is crucial for minimizing delivery errors in cargo transportation, hence safeguarding the interests of both buyers and sellers [1,2,3].

Traditionally, ship draft is mainly measured by manual observation [4]. It typically involves the utilization of yachts, stanchions, and ladders to observe the ship’s six draft marks, aiming to achieve an observation angle as parallel to the water surface as possible [5]. While manual visual observations can yield high precision under static water conditions, they are susceptible to various factors such as parallax errors, limited visibility of draft marks, and diverse weather conditions. These factors often result in significant discrepancies between the actual ship draft and the observed draft [6]. To mitigate the challenges associated with manual observation, sensor-based automated draft reading approaches have been proposed [7,8,9]. By utilizing customized sensors to collect pressure and distance data of the ship, they could measure a ship’s draft in an indirect manner. However, these sensors may also be affected by ambient noise from the marine environment and inherent sensor noise. Moreover, sensor-based methods often entail high costs and challenging installations, making them inapplicable to practical implementation.

On the other hand, image processing methods using advanced technologies offer a new direction for ship draft reading. They enable direct observation of the ship’s draft [10,11] and are more suitable for practical applications due to their low cost, simple operation, and strong reproducibility. Early research in this field employed machine vision techniques to determine the values of draft marks intersecting with the waterline [12]. Typically, the acquired images are initially cropped to attain the target area while eliminating environmental interference. Subsequently, traditional image processing methods are applied to extract features from the cropped draft image, thus identifying the waterline’s position. Finally, postprocessing techniques such as character recognition and draft calibration are employed to determine the intersection position of the waterline and the draft marks, facilitating further measurements of a vessel’s draft. For image processing methods, Tsujii et al. [13] detected draft marks with morphological operations and located the position of the waterline with Canny edge detection. Ran et al. [14] adopt the Canny edge detection algorithm to extract the waterline containing the contours from the image, then utilize the Hough transform to detect the waterline. However, these image processing methods pose challenges in adapting to diverse and complex scenarios, often requiring postprocessing efforts tailored for different situations. In addition, their reading accuracy often fails to meet practical application standards.

**Figure 1:**
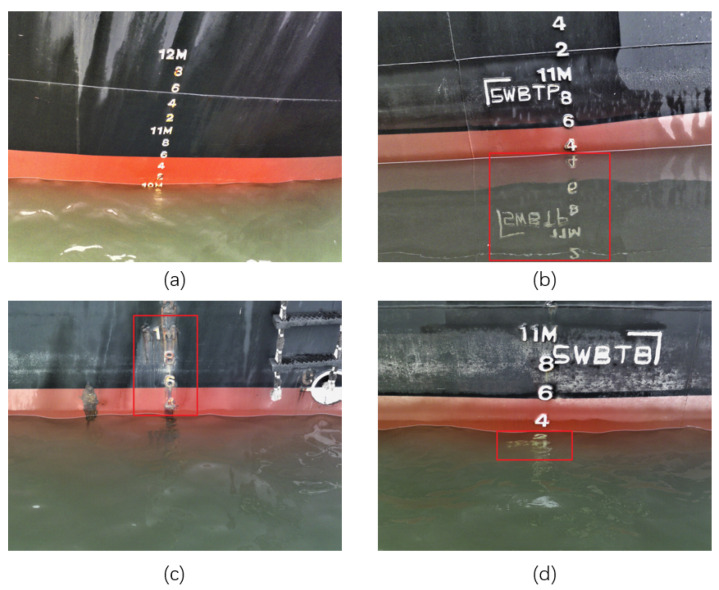
(**a**) Normal draft image without the issues of surface reflection or character erosion. The reflection of the part selected by the red box in (**b**) is very obvious, which may confuse the reflection during target detection and affect the accuracy of water segmentation. The characters in (**c**) have serious corrosion, which affects the recognition accuracy of target detection. The characters in the red box in (**d**) are submerged, but the water body is relatively clear, so they are still visible in the image and are recognized by the algorithm.

The emergence of deep learning (DL)-based image processing techniques has alleviated the aforementioned challenges. By integrating DL algorithms with RGB ship draft images, significant strides have been made in draft reading, presenting superior performance in terms of accuracy and efficiency [15]. For instance, Wang et al. [16] use mask R-CNN to segment the draft markers and water from the image, while UNet and ResNet are adopted for waterline detection and character recognition respectively. This visual information collectively contributes to precise draft readings. Li et al. [17] propose U^2^-NetP, incorporating coordinate attention for semantic segmentation and achieving 96.47% accuracy for waterline segmentation. In addition, their method is combined with a lightweight YOLO-v5n network architecture to detect the ship draft characters and reaches 98% of *mAP*_0.5. Qu et al. [18] propose a multitask learning network named MTL-VDR for draft recognition and waterline segmentation, enhancing both reading efficiency and accuracy. Despite these advancements, the current DL-based algorithms coupled with RGB ship draft image collection sensors still face challenges in real-world implementation. As shown in Figure 1, the water surface reflections and character erosion problems would confuse the model with additional or unclear characters, leading to an adverse influence on ship draft reading performance. Besides, RGB sensors are easily affected by environmental and illumination variations, complicating model training with diverse image inputs.

Multispectral images contain discriminating spectral information crucial for object recognition, effectively supplementing the spectral data absent in RGB images [19]. Consequently, in various domains, the integration of multispectral images with RGB data has been researched to achieve a more comprehensive understanding of target objects. For instances, Barrero et al. [20] combine the texture information from the RGB image with the reflectance information given by a multispectral image to obtain fused RGB-MS images with better weed identification features. Zhang et al. [21] propose a novel feature fusion approach that exploits the complementary and consistent balance of multispectral features by adding a dedicated module into the network architecture, iteratively fusing and refining each spectral feature. Soroush et al. [22] integrate each RGB channel with the NIR channel based on visual saliency mapping, demonstrating improved classification results with the fused NIR/RGB data when applied to deep convolutional neural networks. Furthermore, some researchers leverage the spectral reflectance characteristics of multispectral images in tasks such as water feature extraction and remote sensing segmentation, achieving promising outcomes [23,24,25]. The notable success of previous studies underscores the value of multispectral images (MSI) as complements to RGB data in model input, enhancing the performance of DL-based image recognition algorithms across diverse tasks. However, little research has been conducted focusing on the application of MSI in ship draft reading tasks.

Therefore, to effectively distinguish water surfaces and ship bodies while minimizing the impact of optical environmental factors such as water surface reflections, we propose a framework to integrate NIR and RGB information in draft reading tasks in this study. Firstly, we establish a dual-branch draft reading backbone, namely Band Information Fusion (BIF), to combine the strengths of both types of images. Specifically, it integrates features from both RGB and NIR images by sending them into the two branches of model respectively. Besides, to integrate multiple branches of information, we also design a cross-fusion module (CFM) to unite the parallel MSI-RGB outputs of rich texture details and semantic features. Then, the final fusion feature is obtained to be fed into the multitasking decoder to output the mask of the water body and the character recognition results. Finally, we utilize the Hough transform to fit the waterline and obtain the coordinates of the locations of the waterline, which are combined with the character recognition results to calculate the draft readings using the perspective correction formula. Our main contributions could be summarized as follows:

- This paper innovatively combines NIR and RGB images for automatic draft reading, leveraging their complementary spectral information to mitigate the impact of water surface conditions in draft reading tasks.
- A dual-branch backbone BIF is introduced to extract pairs of information from RGB and NIR images, serving multiple downstream tasks such as waterline segmentation and character recognition.
- Compared with previous research, our method achieved the best results in both waterline segmentation and draft detection tasks, with a *mAP* of 99.2% and *mIoU* of 99.3%, respectively. Additionally, our draft reading error is less than 0.01m compared with the ground truth, achieving the highest accuracy among all the evaluation methods.

## 2. Materials and Methods

### 2.1. Materials

**Figure 2:**
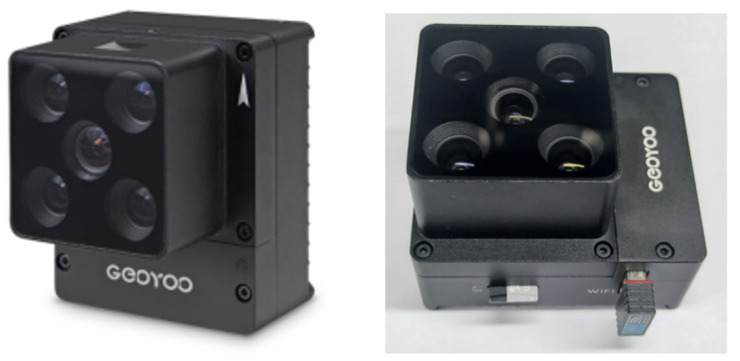
MS400 series multi-spectral camera.

**Figure 3:**
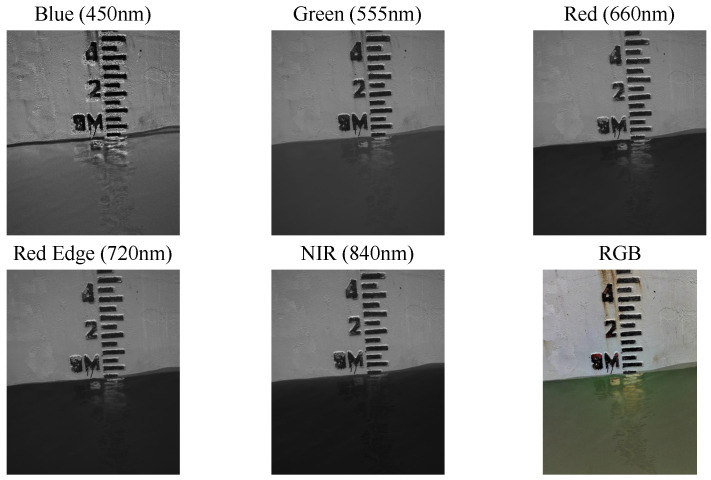
Five band images (Red, Green, Blue, Red-Edge, NIR) and RGB image obtained by cameras.

In this work, the GEOYOO MS400 series multispectral camera (GEOYOO, Changchun, China) was selected as the imaging acquisition device for images (see Figure 2). This type of camera is cost-effective and could synchronously acquire spectral images across multiple wavelengths, including blue band (450 nm), green band (555 nm), red band (660 nm), red edge band (720 nm), and NIR band (840 nm). The spectral information of the MS400 series multispectral camera is displayed in Table 1. The device suite is also equipped with an automatic gray board capture function, facilitating the acquisition of more precise reflectance data when the REF-JPG format is chosen for image storage, thereby guaranteeing a higher level of image quality. The five band images captured by the multispectral camera are illustrated in Figure 3.

The sensitivity of water reflectance varies among different spectral bands, resulting in its color manifestation as deep black when using NIR band (840 nm) information. Meanwhile, metallic surfaces exhibit increased luminescence within the 500 nm to 1000 nm spectral range, enhancing the contrast between vessels and the aquatic environment in captured images. As the wavelength increases, the reflectivity of water decreases, indicating that the difference between water and other objects in multispectral images becomes more discriminating. Consequently, we chose the highest wavelength (840 nm) supported by the camera along with RGB information for dataset construction.

**Figure 4:**
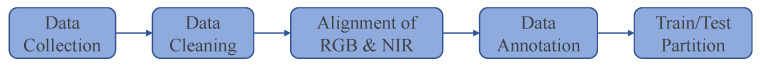
The overall process of dataset construction.

Based on the above camera system, we composed a multispectral dataset for ship draft reading. The construction of this dataset follows a systematic process as shown in Figure 4. Firstly, we collected images from the Huanghua Port, which is operated by China Coal Research Institute Corporation. Each captured image consists of both RGB data and NIR information (at 840 nm wavelength). The resolutions of RGB images and NIR image are 3280 × 2464 and 1280 × 1080, respectively. For image details about the ships in our dataset, the ship color schemes mainly consist of orange and black and orange and blue ships with white characters, as well as white ships with black characters. Besides, all ship images in our dataset adopt national standards for draft levels (such as 2, 4, 6, 8, xxM). It is notable that with all the images captured in calm wind and wave environments, particular scenarios (e.g., wave ripples and larger waves causing longitudinal and transverse deviation of the ships) are not included in our dataset.

**Figure 5:**
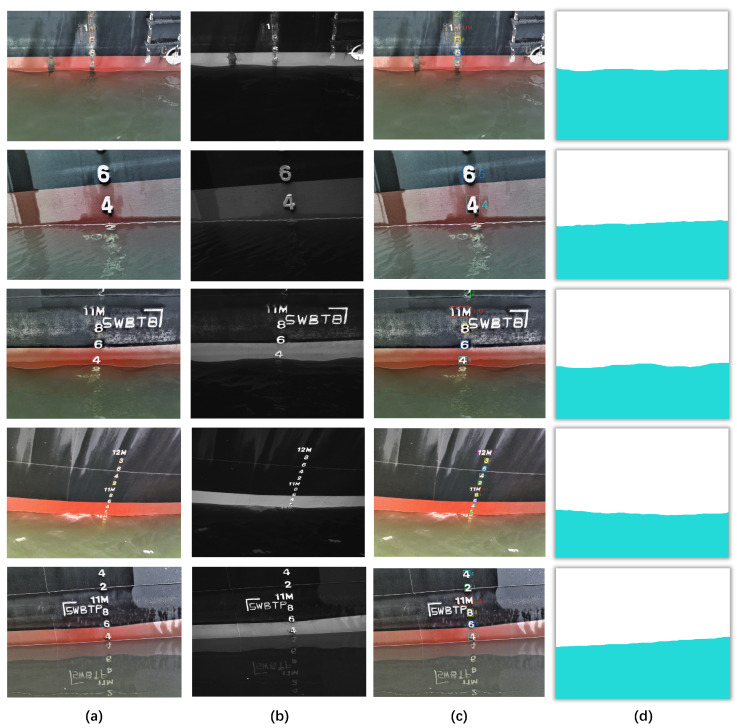
Some examples from our dataset: (**a**): Original images of the ship. (**b**): NIR images (840 nm) of the ship. (**c**): Visualized object detection labels. (**d**): Visualized segmentation labels.

To ensure data quality, we cleaned the images to eliminate any undesirable factors such as tilt, occlusion, or blur that may have resulted from handheld shooting. Eventually, we obtained a dataset comprising 524 images. Some examples of the processed images are displayed in Figure 5. To ensure alignment with the annotation data across all bands within the true RGB images, the NIR images are first rescaled as four-layer images to match the resolution of RGB images. Subsequently, for dataset annotation, we employed the LabelMe tool for mask annotation of aquatic areas in the RGB—NIR images and utilized the MakeSense tool for detecting and annotating frames of ship draft characters in the RGB images. Furthermore, to facilitate model training and validation, we randomly partitioned the dataset into training and validation subsets following an 8:2 ratio.

### 2.2. Methodology

**Figure 6:**
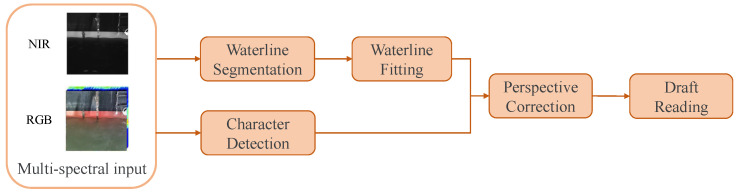
The overall process of draft reading.

The ship draft reading framework proposed in this paper is depicted in Figure 6. Given the multispectral image inputs, we first utilize the proposed Band Information Fusion backbone network combined with the existing heads to obtain the waterline segmentation and character detection results (see Section 2.2.1). Then, the waterline segmentation result is employed to obtain the fitted waterline (see Section 2.2.2). Finally, the ship draft reading is accomplished by performing perspective correction on the waterline and character detection results (see Section 2.2.3).

#### 2.2.1. Band Information Fusion Framework

**Figure 7:**
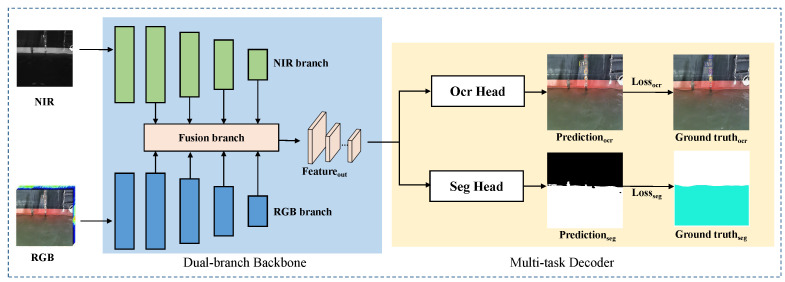
Overview framework of the image-processing-based automated draft reading methods.

The water and ship could be clearly discriminated utilizing multispectral image information, while RGB image provides more color information for character recognition. To leverage the benefits of both NIR and RGB data simultaneously and achieve improved detection and segmentation accuracy, this paper introduces a novel Band Information Fusion (BIF) backbone for data fusion. This backbone could be integrated with existing segmentation and detection heads to perform waterline extraction and character recognition respectively. As illustrated in Figure 7, the RGB image and NIR information are processed independently by two branches of the model. Following multimodal feature alignment and fusion, semantic features are captured at multiple spatial levels. These features are sent into the segmentation head and the detection head to generate the waterline masks and detection frames of the draft characters.

**Figure 8:**
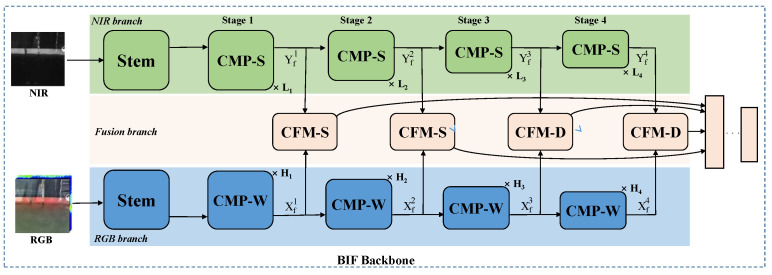
Overview of our BIF backbone. The specific architecture of our novel Band Information Fusion backbone, where $L_{n}$ and $H_{n}$ represent the number of layers of modules stacked.

The overall architecture of the proposed Band Information Fusion backbone is illustrated in Figure 8. In terms of data input, two modalities are fed into the model in parallel, and the intrinsic knowledge is explored through two branches respectively. The two-branch backbone could be divided into four stages, with a stem layer inserted at the beginning of the first stage. In addition, a cross-fusion module (CFM) is designed to unite the parallel NIR-RGB outputs containing rich texture details and semantic features, acting as a bridge to merge the complementary information from the two modalities. Finally, all the multi-level fusion features are obtained and subsequently fed into the multitasking decoder.

**Figure 9:**
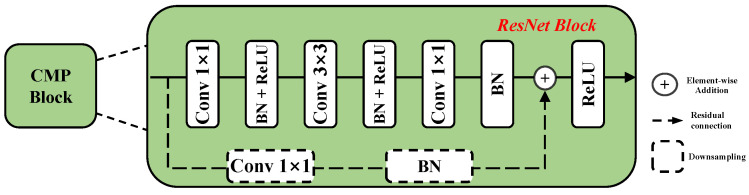
The architecture of Cross-Modality Process basic module.

**Overview of branch structure**: In this work, we use the modified ResNet [26] as the dual-branch basic structure. Given an input with a size of $\frac{H}{N}\times \frac{W}{N}$ (where *N* represents the image channel), the resolution is diminished to $\frac{H}{4}\times \frac{W}{4}$ following the stem layer, as shown in Figure 8. It includes a combination of convolution, Batch Normalization, Rectified Linear Unit, and max pool. Subsequently, the features are processed through four stages, resulting in $Y_{f}^{1}\left(X_{f}^{1}\right),Y_{f}^{2}\left(X_{f}^{2}\right),Y_{f}^{3}\left(X_{f}^{3}\right)$ and $Y_{f}^{4}\left(X_{f}^{4}\right)$ features. The resolutions of these four features are halved in sequence, specifically $\frac{1}{4},\frac{1}{8},\frac{1}{16}$, and $\frac{1}{32}$, and the channels increase in order of C, 2C ,4C, and 8C. Figure 9 illustrates the basic Cross-Modality process spectral (CMP-S) and Cross-Modality process window (CMP-W) blocks of the ResNet-18 and ResNet-50 at each stage. The structure of the basic module is composed of stacked convolutions, BN and RELU, where the input feature is added to the main branch output feature via a shortcut and then passed through a ReLU activation function to enhance the model’s nonlinearity. At the beginning of each stage, the residual connection includes an embedding convolution and BN to downsample the features. The incorporation of bottleneck architectures and residual connections facilitates the training process. Moreover, to handle the different complexity of two-branch features, the number of basic modules stacked in each stage are $L_{1},\;L_{2},\;L_{3}$ and $L_{4}$ and $H_{1},\;H_{2},\;H_{3}$ and $H_{4}$, respectively, where the number of blocks in the RGB branch is twice that of the NIR branch.

Cross-fusion module: CFM is specially designed to encode detailed features and unite the parallel NIR-RGB outputs. In consideration of the differences among characteristic output of the stages and model parameters, we designed two forms of CFM for shallow and deep fusion separately, namely Cross-Fusion Module Shallow (CFM-S) and Cross-Fusion Module Deep (CFM-D).

**Figure 10:**
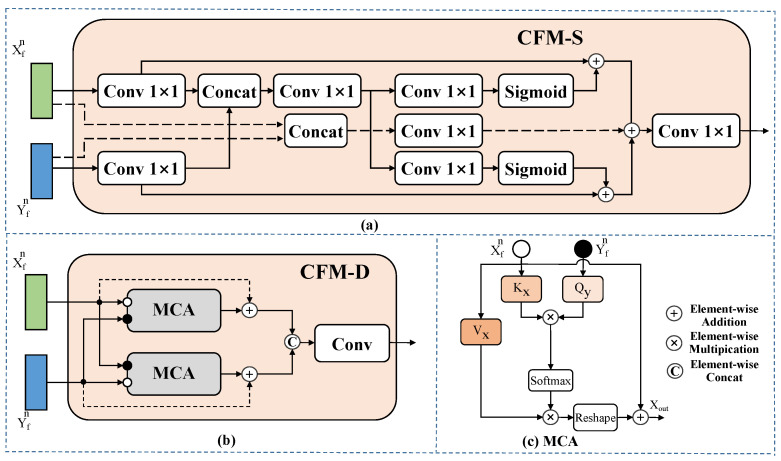
(**a**) The illustration of CFM-S in BIF. (**b**) The illustration of CFM-D in BIF. (**c**) The detailed structure of MCA module.

For CFM-S, as a design of minimal complexity, it is implemented in shallow features without great burden. Furthermore, it could adaptively fuse the two-branch inputs while transmitting detailed features between RGB and NIR pairs, enabling the preservation of the local information to the greatest extent. As shown in Figure 10a, initially, the input features from the dual branches are processed through a 1×1 convolution, respectively. After that, the concat operation combines these two features and forwards them to the next convolution for facilitating interaction among the NIR-RGB pairs. Then, the features are subjected to a 1×1 convolution and a sigmoid function to normalize the pixel values within the range of 0 to 1, which are then multiplied pixel by pixel with the previous features to perform pixel-level reweighting operations. Finally, we employed residual connection to add previous features, aiming to accelerate model optimization and reduce the learning complexity of feature weight maps.

For CFM-D, it is a feature fusion module based on an attention mechanism, capable of selecting beneficial information from the spectral and spatial dimensions to supplement each branch. It is worth noting that the use of a high-parameter Multihead Cross-Attention (MCA) module in the deep 3rd and 4th stages will not introduce excessive computational cost due to the multiple downsampling procedures. As depicted in Figure 10b, CFM-D receives dual-branch features and employs the pairs of features as the query input for the two parallel MCA modules in a cross-attention manner. MCA could learn feature mapping relevance and calculate spatial correlation for pairs of inputs. Given the inputs $X_{f}^{n},Y_{f}^{n}\in R^{HW\times C}$ with stage n, height H, width W, and the number of channel dimensions C, where f stands for fusion, the MCA is expressed as

(1) $$Q_{y}=Y_{f}^{n}w_{y}^{q},K_{x}=X_{f}^{n}w_{x}^{k},V_{x}=X_{f}^{n}w_{x}^{v},$$

(2) $$M_{\mathrm{out}}=\mathrm{softmax}\left(\frac{Q_{y}K_{x}^{T}}{\sqrt{d_{k}}}\right),X_{out}=V_{x}M_{out},$$

where $X_{out}$ is the fused results, $w_{y}^{q},w_{x}^{k},w_{x}^{v}$ correspond to the learnable weights of the query Q, key K, and value V. The activation function softmax can normalize the correlation weights. $d_{k}$ indicates the dimension of $K_{x}$ that is used to scale the matrix, and ${\left(\cdot \right)}^{T}$ is the matrix transpose. In addition, $M_{out}\in R^{HW\times HW}$ denotes the mappings between the pairwise NIR-RGB inputs over multiple bands information. Afterwards, the fused dual-branch embedding is ultimately obtained through simple concatenation and convolution.

#### 2.2.2. Waterline Fitting

After obtaining the water body mask and the character recognition results, we use the Hough transform method to extract the contour of the water mask and fit the curve with the contour of the boundary between water and ship. Specifically, each pixel in the image is transformed into a parametric coordinate system. The intersection of multiple straight lines in this system corresponds to a straight line in the image space, namely the boundary line of the water body we are looking for. Aiming to avoid inaccurate readings resulting from the sea level fluctuations caused by wind and waves, we take the average y-axis coordinates of all pixels on the waterline as the position coordinates of the waterline.

#### 2.2.3. Perspective Correction and Reading

**Figure 11:**
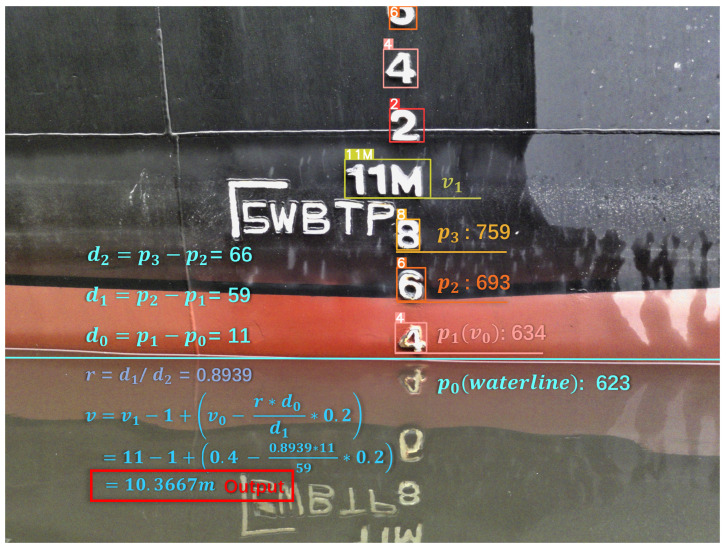
Illustration of draft calculation. Firstly, the ratio of the vertical distance (*r*) between each character is computed as the correction factor for the perspective problem. Secondly, we determine the integer digit of the readings based on the classification result ($v_{1}$) of the character with “M”. In the following step, we calculate the decimal place of the readings via the vertical position of the waterline, the distance of the character closest to the waterline ($v_{0}$), and its numerical category. In the end, the final readings are further obtained by calculating readings with the correction factor.

After detecting the draft characters and extracting the coordinates of the waterline position, the draft reading can be calculated according to the reading formula. It is notable that the perspective problem occurs since the shooting angle is not always aligned with the ship. To solve this problem, we need to adopt the ratio of the vertical distance (*r*) between each character as the correction factor. Taking Figure 11 as an example, considering all the calculation steps, 5 positions and 3 vertical distances are needed to be determined first. The first position ($p_{0}$) is the position coordinates of the waterline. The other four positions include the positions of three consecutive characters from bottom to top ($p_{1}$, $p_{2}$, $p_{3}$) and the position of the character with the letter “*M*” that is the closest to the waterline ($v_{1}$). Then, three distances are calculated as follows in Equation (3):

(3) $$\begin{array}{c} d_{0}=p_{1}-p_{0},d_{1}=p_{2}-p_{1},d_{2}=p_{3}-p_{2} \end{array}$$

where $d_{1}$ and $d_{2}$ denote the complete character spacing of two pairs of characters, so the perspective coefficient is $r=d_{1}/d_{2}$.

In the end, the specific readings are determined by the classification results of the character detection $v_{0}$ and $v_{1}$. Here, $v_{0}$ is the classification result of the character closest to the waterline, and $v_{1}$ is the classification result of the character with “*M*” that is the closest to the waterline. Since the real distance between the two characters is 0.2 m, the formula for the final reading result *v* is given in Equation (4):

(4) $$\begin{array}{c} v=v_{1}-1+\left(v_{0}-\frac{r*d_{0}}{d_{1}}*0.2\right) \end{array}$$

## 3. Results

### 3.1. Evaluation Metrics

To evaluate the effectiveness of our draft reading results comprehensively, we evaluate our method in dimensions of detection, segmentation, and ship draft readings using the following evaluation metrics: mean Average Precision (*mAP*), mean Intersection over Union (*mIoU*), and Mean Absolute Error (*MAE*).

*mAP* is a crucial metric used to measure the performance of object detection algorithms, obtained by averaging the AP of detection boxes across all categories. The equation defining *mAP* is formulated as follows:

(5) $$\begin{array}{c} mAP=\frac{1}{n}\sum_{i=1}^{n}\frac{1}{c}\sum_{k=1}^{c}P_{k}R_{k} \end{array}$$

where $R_{k}$ is the proportion of the correctly detected boxes number to the ground truth boxes number. $P_{k}$ is the ratio of the correctly detected box numbers to the total detected box numbers. *c* is the number of categories detected. *n* is the total number of object categories.

*mIoU* is a commonly used segmentation metric for measuring the degree of overlap between the region predicted by the model and the truth labels. It is the intersection of the predicted area and the actual area divided by the union of these two areas, with the results obtained for all categories being averaged. The calculation formula is illustrated in Equation (6)

(6) $$\begin{array}{c} mIoU=\frac{1}{n}\sum_{i=1}^{n}\frac{TP}{FN+FP+TP} \end{array}$$

where *TP* is the number of positive classes predicted to be positive, and *FP* is the number of negative classes predicted to be positive. *FN* is the number of positive classes predicted to be negative.

Finally, *MAE* (Mean Absolute Error) is used to measure the difference between the draft line obtained by manual readings and our automatic methods. The manual reading means the average result of multiple careful readings of the waterline position on the images by experts. This comparison directly reflects the actual feasibility of our proposed automatic methods.

### 3.2. Experimental Setup

In this study, all experiments are conducted on a platform with the Ubuntu 20.04 operating system, NVIDA RTX2080T with 12 GB RAM (NVIDIA, Santa Clara, CA, USA), and Intel Core i5-13600k with 32 GB RAM (Intel, Santa Clara, CA, USA). The software platform is Pytorch 1.12.1 based on Python 3.8.0.

We validated the effectiveness of our method based on two detection methods (YOLOv5 and YOLOv8) and two segmentation methods (DeepLabv3+ [27] and UPerNet [28]) by replacing the original backbone (ResNet-50) with our proposed multiscale two-branch backbone. The hyperparameter settings used for training are shown in Table 2, including epoch, batch size, learning rate, and optimizer.

### 3.3. Algorithm Results

In this section, we verify the effectiveness of the proposed backbone on detection, segmentation, and manual reading comparison. The validation sets contain a total number of 105 images. Based on the constructed datasets, we compared our backbone performance with the original ResNet-50 backbone in four segmentation and detection methods. When choosing ResNet-50 as the backbone, the utilization of four band images (RGB+NIR) represents concating RGB and NIR information as a four-channel input. We also compare the draft reading results of three segmentation+detection methods with the manual readings. All the experiment results demonstrate that by introducing multispectral data and designing an effective feature fusion network to fully utilize RGB and NIR information, ship draft reading performance can be effectively improved.

**Analysis of character detection results**: Table 3 shows the comparison of detection performance between YOLOv5, YOLOv8, and ours. The *mAP* achieved by YOLOv5 with ResNet-50 backbone using RGB image input is 94.1%. When replacing inputs with the RGB and NIR image integration, the *mAP* score increases to 95.0%, with an improvement of 0.9%. It is evident that the utilization of multispectral information significantly enhances the results. With further replacement with our backbone, the YOLOv5 model attains a notable *mAP* value of 95.9%, presenting a substantial improvement in detection performance. For YOLOv8 based methods, better results could be achieved with basic settings (using ResNet-50 backbone and RGB image input). Similarly, the additional utilization of NIR data and our backbone lead to superior performance, respectively. And the optimal result is obtained on the setting of YOLOv8 with our backbone using RGB+NIR input data, with *mAP* improving up to 99.2%. These results by different methods and input sources prove the effectiveness of utilizing multispectral information with our architecture design in the detection task.

**Analysis on waterline segmentation results**: The segmentation results of DeepLabv3+, UPerNet, and ours are presented in Table 4. A *mIoU* score of 98.0% is achieved using the DeepLabv3+ model with RGB image input and ResNet-50 backbone. Upon transitioning to using RGB+NIR images as input, the *mIoU* score increased to 98.3%, proving that the integration of NIR information could bring additional improvement in the segmentation task. When replacing ResNet-50 with our backbone, the *mIoU* score further rises to 99.0%, with an enhancement of 0.7%. The implementation of our backbone effectively boosts the segmentation results. A similar experiment conducted with the UPerNet model also shows that the use of RGB+NIR inputs outperforms the use of pure RGB images. In comparison with ResNet-50, the employment of our backbone results in a marked increase for the segmentation task, reaching *mIoU* of 99.3%. In conclusion, combining RGB+NIR inputs and BIF backbone also takes effect in the segmentation of the waterline.

**Figure 12:**
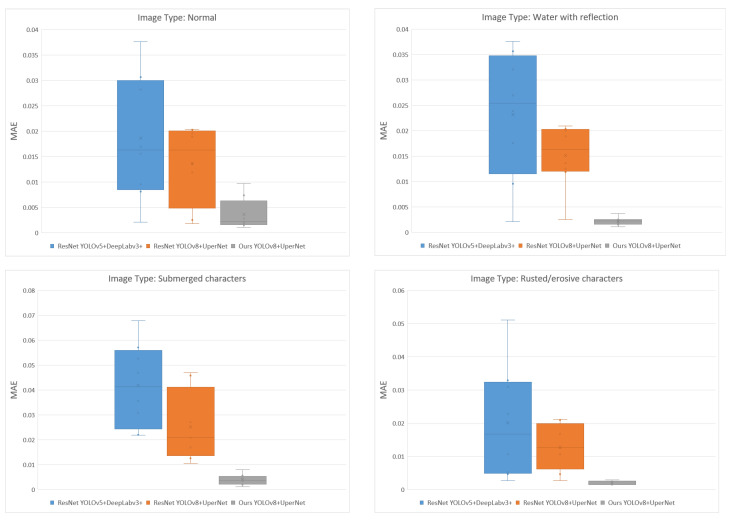
Box plot analysis of different methods in the draft reading task.

**Analysis on draft reading results**: For reading performance compared with the manual reading approach, waterline images in validation sets are classified into four types, including waterlines that suffer from different degrees of environmental issues, namely normal, water with reflections, submerged characters, and rusted characters. The utilized segmentation+detection models for draft reading performance comparison contain original YOLOv5 + DeepLabv3+, original YOLOv5 + UPerNet, and our backbone-based YOLOv8 + UPerNet. The results are shown in Table 5. We set the manual readings as the baseline and *MAE* as the validation indicator. For normal waterline images, the *MAE* is 0.021 m using YOLOv5 with DeepLabv3+ and 0.013 m using YOLOv8 combined with UPerNet. The minimum *MAE* obtained using our backbone method is 0.007 m. For images with water reflections, our method has an error of only 0.003 m. Compared with the original backbone, it reduces the *MAE* by at least 0.01 m, indicating that our proposed backbone effectively filters reflections to obtain more accurate readings. For waterline images with submerged characters, an error of 0.051 m is exhibited when employing the YOLOv5 + DeepLabv3+, whereas utilizing YOLOv8 + UPerNet reduces the *MAE* to 0.031 m. A significant enhancement in *MAE* is achieved by substituting the ResNet backbone with our backbone, resulting in the lowest *MAE* of 0.005 m. For the images with rusted characters, our method also has a minimum *MAE* of only 0.002 m. It can be seen that the combination of multispectral information and special design BIF backbone further narrows down the reading errors in comparison with the methods using only RGB data in all four types of waterline images. A statistical comparison is also presented in Figure 12. For different kinds of images, the distribution of each method can be seen from this figure. The *MAE* results of our methods are the lowest among the three algorithms. Additionally, considering all results, our method achieves the smallest variance, seen from the size of the box, proving its superiority over other methods.

## 4. Conclusions

In this paper, we innovatively introduce multispectral images as data input in character detection and waterline segmentation algorithms for the ship draft reading task, in addition to a specially designed fusion backbone, further improving the model performance and robustness in special environments. First, we capture NIR and RGB images in the field by MS400 and construct a multispectral dataset. To leverage the advantages of NIR and RGB images, we design a dual-branch backbone (BIF) to merge the features extracted from both types of images. Coupled with segmentation and detection heads, the features extracted by the proposed backbone network could simultaneously handle waterline segmentation and character detection tasks. On our self-constructed multispectral draft image dataset, our method achieves 99.2% *mAP* for waterline segmentation and 99.3% *mIoU* for draft detection tasks. Finally, in the draft reading task, our *MAE* is less than 0.01 m in all four types of waterline images, achieving superior results that are the closest to human observation compared with other methods.

## Figures and Tables

**Table 1:** Spectral information of the multispectral camera.

Band No.	Name	Center Wavelength	Bandwidth
1	Blue	450 nm	35 nm
2	Green	555 nm	25 nm
3	Red	660 nm	22.5 nm
4	Red Edge	720 nm	10 nm
5	NIR	840 nm	30 nm

**Table 2:** The training parameters of each network.

Model	Epoch	Batch Size	Learning Rate	Optimizer
Our YOLOv5	100	8	0.001	ADAM
Our YOLOv8	100	8	0.1	ADAM
Our DeepLabv3+	100	8	0.005	SGD
Our UPerNet	100	8	0.02	SGD

**Table 3:** Experiments of different methods in character detection task.

Backbone	Input Type	Model	*mAP* (%)
ResNet-50	RGB	YOLOv5	94.1
ResNet-50	RGB	YOLOv8	96.7
ResNet-50	RGB + NIR	YOLOv5	95.0
ResNet-50	RGB + NIR	YOLOv8	97.9
Ours	RGB + NIR	YOLOv5	95.9
Ours	RGB + NIR	YOLOv8	99.2

**Table 4:** Experiments of different methods in waterline segmentation task.

Backbone	Input Type	Model	*mIoU* (%)
ResNet-50	RGB	DeepLabv3+	98.0
ResNet-50	RGB	UPerNet	98.4
ResNet-50	RGB + NIR	DeepLabv3+	98.3
ResNet-50	RGB + NIR	UPerNet	98.9
Ours	RGB + NIR	DeepLabv3+	99.0
Ours	RGB + NIR	UPerNet	99.3

**Table 5:** Comparison of draft reading by different methods using the *MAE* metric.

Image Type	ResNet		Ours
	YOLOv5 + DeepLabv3+	YOLOv8 + UPerNet	YOLOv8 + UPerNet
Normal (11 images)	0.021 m	0.013 m	0.007 m
Water with reflection (57 images)	0.023 m	0.014 m	0.003 m
Submerged characters (16 images)	0.051 m	0.031 m	0.005 m
Rusted/erosive characters (21 images)	0.034 m	0.018 m	0.002 m

## Data Availability

The datasets presented in this article are not readily available because the data are part of an ongoing study. Requests to access the datasets should be directed to the email zb_ccric@163.com.
